# Duct Stenting vs. Modified Blalock-Taussig Shunt: New Insights Learned From High-Risk Patients With Duct-Dependent Pulmonary Circulation

**DOI:** 10.3389/fcvm.2022.933959

**Published:** 2022-06-23

**Authors:** Nathalie Mini, Martin B. E. Schneider, Boulos Asfour, Marian Mikus, Peter A. Zartner

**Affiliations:** ^1^Department of Cardiology, German Pediatric Heart Center, University Hospital of Bonn, Bonn, Germany; ^2^Department of Pediatric Cardiac Surgery, German Pediatric Heart Center, University Hospital of Bonn, Bonn, Germany; ^3^Department of Anaesthesiology and Intensive Care Medicine, University Hospital Bonn, Bonn, Germany

**Keywords:** duct stenting, mBT shunt, sinusoid blood flow, duct-dependent pulmonary circulation, ductal curvature index, tortuosity index, pulmonary atresia (PA)

## Abstract

**Background:**

As no data were available on the comparison of outcomes between modified Blalock-Taussig shunts (MBTs) vs. duct-stenting (DS) in patients with pulmonary atresia (PA) and an increased ductal tortuosity and in patients with pulmonary atresia and intact septum (PA-IVS) with right ventricle-dependent coronary circulation (RVDCC), we aimed to perform a single-center retrospective evaluation.

**Methods:**

Between 2010 and 2019, 127 patients with duct-dependent pulmonary circulation (DDPC) underwent either MBTs (without additional repairs) (*n* = 56) or DS (*n* = 71). The primary endpoint was defined as arriving at the next planned surgery (Glenn or biventricular repair) avoiding one of the following: (1) unplanned surgery or unplanned perforation of the pulmonary valve (PVP) with a stent, (2) procedure-related permanent complications, and (3) death. Two subgroups were considered: (1) patients who had a ductal curvature index (DCI) >0.45 (*n* = 32) and (2) patients with PA-IVS and RVDCC (*n* = 13). Ductal curvature index (DCI) was measured in all the patients to assess the tortuosity of the ducts. Patients with DCI >0.45 were considered as being in a high-risk group for the duct-stenting; a previous study showed that the patients with a DCI < 0.45 had a better outcome when compared with those with a DCI> 0.45.

**Results:**

The primary outcome was achieved equally in the two groups (77.5% in DS, 75% in MBTs). Hospital deaths, need for ECMO, and the occurrence of major complications was more frequent in the group with MBTs with an Odds Ratio (OR) of 5, 0.8, and 4, respectively, and a 95% Confidence Interval (*CI*) 1.1–22.6, 0.7–0.9, and 1.6–10.3, respectively, and a *P*-value < 0.05. For the two subgroups, the primary outcome was achieved in 64% of patients with a DCI >0.45 who received MBTs compared to 20% in those with DS (OR 3.5, 95% *CI* 1.2–10, *P* 0.005). While 74.1% of the patients with PA-IVS and RVDCC after DS had achieved the primary outcome, all patients with MBTs had an impaired outcome (OR 3.5, 95%*CI* 1–11.2, *P* 0.004).

**Conclusion:**

MBTs showed a better outcome in patients with tortuous ducts compared to DS. DS seems to be superior in patients with DDPC with DCI <0.45 and patients with PA-IVS with RVDCC.

## Introduction

In patients with pulmonary atresia (PA) and a duct-dependent pulmonary circulation maintaining pulmonary blood flow may be achieved either by the surgical creation of a modified Blalock Taussig shunt or the percutaneous ductal stenting. Although surgical techniques have advanced in recent years (1), achieving a biventricular repair with DDPC is still challenging in the neonatal period (2–5). The efficacy of DS as an alternative to MBTs has been documented in multiple studies (6, 7). The mortality rate, need for ECMO, and procedure-related complications were reported to be higher in the MBTs group compared to the DS group due to post-operative hemodynamic instability (8–10). Thus, in patients with PA and DDPC, the DS has become the preferred method of choice in some centers. However, no study has compared the outcome between the two procedures in patients with highly tortuous ducts in which the DS is challenging and the outcome can become inadequate. In an earlier study from 2021, (11) we were able to show that the patients with a ductus curvature index (DCI) above 0.45 belong to a high-risk group for proper stent placement, which resulted in inadequate palliation to the next planned operation. Data on the outcome of this high-risk group with MBTs is not published yet. So, we focused on this group as well as on the comparable complex group of the patients with PA and an intact ventricular septum with a coronary sinusoid to identify the preferable palliative procedure in these two groups.

## Patients and Methods

We performed a single-center retrospective cohort analysis. All patients with PA and DDPC undergoing either DS (*n* = 71) or MBTs (without additional repair *n* = 56) between 2010 and 2019 were recruited and the outcome between DS and MBTs were compared. To evaluate the severity of ducts-tortuosity the ductal curvature index (DCI) was measured in all patients who received either diagnostic or interventional heart catheterization (*n* = 88). DCI was measured using the following formula adopted from a previous study (8): DCI = L2-L1/L2 (L1 represents the straight short distance between duct origin from the aorta and duct insertion onto pulmonary arteries, L2 represents the entire length of the duct between the aortic origin and the insertion of the duct onto PA.

Based on our previous study published in 2021 (9) high-risk patients for stenting were defined as patients having DCI equal or higher than 0.45.

Additionally, two subgroups were established to study more specifically and for the first time the outcome between DS and MBTs in special cases. The first subgroup includes the patients who belong to the high-risk group for stenting (9) with DCI >0.45 (*n* = 32) and the second includes the patients who have PA-IVS and coronary sinusoids (*n* = 13). All patients with an additional pulmonary blood supply like patients with PA and major aorta pulmonary collateral Arteries (MAPCAs) were excluded from this study, as well as patients with pulmonary stenosis who have significant antegrade pulmonary perfusion. All patients who received MBTs with additional repair were also excluded from the analysis to focus on the real impact of MBTs and DS on the outcome.

The primary outcome of this study is defined as the successful bridging of patients, who received either DS or MBTs, to the next planned procedure (Glenn or biventricular repair) with freedom from the following: (1) unplanned surgery or perforation of the pulmonary valve (PVP) with a stent, (2) permanent procedure-related complications, and (3) death.

The secondary outcome is defined as the need for ECMO, the occurrence of procedure-related complications, the need for pulmonary artery plasty (PA-plasty) at the time of the next surgery, and the need for additional stent-implantations into the stented duct or into the MBTs.

Additional comparison between the two procedures was performed for the two subgroups (patients with DCI >0.45 and patients with PA-IVS and coronary sinusoid). Hospital deaths, major procedure-related complications, the need for ECMO, and the need for PA-plasty at the time of surgery was documented. Total lower lobe index (TLLI), Nakata and McGoon index were used as pulmonary parameters to estimate the growth of pulmonary arteries at the closest time before the next planned procedure.

### Statistical Analysis

All statistical analyses were performed using SPSS version 22 (IBM). Continuous variables were reported as means ± standard deviations (*SD*) and categorical variables as count (percentage). The non-paired Student's *t-*test was used to compare the means of continuous variables between the two different categories, while paired *t-*test was applied for comparing the means of the pulmonary parameter before and after the two procedures for the whole cohort. The Chi-square test was used for comparing categorical variables. Odds ratios (ORs) ± 95% confidence intervals (95% *CI*) for the following parameter were calculated to assess any differences between DS and MBTs: hospital deaths, procedure-related complications, need for ECMO, the need for unplanned surgery/PVP with a stent, the need for PA-Plasty, the need for additional stent implantation in DS or MBTs, and the outcome. A *P*-value of 0.05 was set as the threshold for clinical significance. Kaplan-Meier survival curve of the two different procedures was performed.

### Ethical Statement

The study complies with the declaration of Helsinki (as revised in 2013). Owing to a purely retrospective study design, using available institutional clinical records, with an absence of impact on the management of the patients included and completely anonymous data presentation, informed consent of the subjects (or their parents) and ethical approval have not been obtained.

## Results

A total of 127 patients with truly DDPC underwent either DS (71 patients) or MBTs (56 patients). The stenting was performed retrograde in 59 patients and antegrade in 12. At the time of DS mean weight was 3.3 kg (SD 0.68) with a range of 3.2 (2–5 kg). The Mean Age was 18 days (SD 23.18), (range 1–120 days). The ductal curvature index (DCI) was higher than 0.45 in 15 patients with DS. At surgical creation of MBTs mean weight was 3.6 kg, SD 1,1 (range 2.1–7.4 kg), and the mean age was 30.3 days with a SD of 34.8 days (range 1–155 days).

The size of MBTs was 4 mm in nine patients and 3.5 mm in the rest of the patients.

### Mortality and Complications

No intervention or operation-related mortality was reported during the two procedures. About three hospital deaths were reported in three patients (4.22%) 1, 7, and 47 days after DS compared to eight hospital deaths 1, 2, 10, 11, 14, 25, 30, and 39 days after MBTs. Two (2.8%) of hospital deaths after DS were DS-related death, and the third person died due to problems related to pre-maturity. All hospital deaths after MBTs (14.2%) were complication-related deaths after MBTs.

There were five major complications associated with DS compared to 15 associated with MBTs (Table 1). The DS-related complications were stent thrombosis in four and vessel damage with severe neurologic complications in one patient. The MBTs-related complications were shunt thrombosis in six patients, necrotic enterocolitis (NEC) in one patient, renal failure with NEC and intracranial hemorrhage in one patient, pericardial tamponade with a need for ECMO in one patient, ventricular tachycardia with secondary decompensation and need for ECMO in two patients, and desaturation and low cardiac output with a need for ECMO in four patients.

**Table 1:** A summarized overview of diagnoses, complications, and outcomes in this cohort study.

**Description**	**DS**	**MBTs**	**OR**	**95% *CI***	***P*** **-value**
Patients	71	56			
PA IVS	10 (14.1%)	7 (12.5%)			
PA VSD	49 (69%)	49 (87.5%)			
Critical PS IVS	12 (16.9%)				
**Primary outcome**					
Unplanned surgery	11 (15.5%)	3 (5.3%)	1.1	0.5–2.8	0.89
Unplanned PVP with stent	2 (2.8%)	0			
Unplanned glenn	1 (1.4%)	1 (1.8%)			
Unplanned shunt-clips		6 (10.7%)			
Permanent complication	1 (1.4%)	1 (1.8%)			
Hospital deaths	3∧ (4.2%)	8 (14.2%)	5	1.1–22.6	0.017^*^
**Secondary outcome**					
PA-Plasty at glenn time	18 (25.3%)	8 (14.2%)	0.5	0.2–1.2	0.094
At shunt time		11 (19.6%)			
Major complications	5 (7%)	16 (28.5%)	4.05	1.6–10.3	0.001^*^
Need of ECMO	0	9 (16%)	0.83	0.7–0.9	0.000^*^
Unplanned stent implantation	6 (8.4%)	10 (17.8%)	2.1	0.8–5.4	0.049^*^
Unplanned stent dilation or implantation	15 (21.1%)	10 (17.8%)	0.8	0.3–2	0.4
Primary outcome	*54 (77.5%)*	*42 (75%)*	*0.9*	*00.7–1.1*	*0.5*
Impaired outcome	*16 (22.5%)*	14^**^ (25%)			

*OR, odds ratio; 95% CI, confidence interval; PA IVS, pulmonary atresia; IVC, intact ventricular septum; VSD, ventricular septum defect; ECMO, extracorporeal membrane oxygenation; PA-Plasty, pulmonary artery plasty; PVP, perforation of pulmonary valve; ∧two hospital deaths (2.8%) were DS-related deaths, and one was a non-cardiac related death;*

** *Impaired outcome 15, one patient has died due to tumor-related problems*.

* *P-value < 0.05 significant*.

### Primary and Secondary Outcome Results

The primary outcome was achieved in 54 patients undergoing DS compared to 42 patients undergoing MBTs. The need for the ECMO in patients with MBTs was observed in nine while there was no need for ECMO in the DS group. Unplanned surgery or PVP and RVOT-stent had to be performed in 13 patients with DS and in 9 patients with MBTs. PA-plasty at the next planned surgery (Glenn or repair) was necessary in 18 patients who received DS compared to 8 for those with MBTs. The rate of unplanned stent implantation was 10 in the MBTs group compared with 6 in the DS group. The measured pulmonary parameters showed a significant increase in all patients of the two groups in whom the next planned procedure was achieved. Nakata index improved from a mean of 171 before to 286mm^2^/m^2^ after stenting and to 270 mm^2^/m^2^ after MBTs, McGoon index from 1.5 to 2.7 after the DS compared to 2 after MBTs and TLLI from 90 to 169 mm^2^/m^2^ after the DS compared to 191 mm^2^/m^2^ after MBTs.

### High Ductal Curvature Index (DCI >0.45) and Outcome

A total of 32 patients with true DDPC and a DCI of more than 0.45 underwent cardiac catheterization. DS was performed in 15 patients (Table 2). About eight of these patients needed emergency MBTs due to desaturation or due to severe reintervention-related complications (Stent thrombosis *n* = 5, stent dislocation1, severe obstruction 2). The patients (*n* = 17), who received catheterization without intervention, were referred to surgery for surgical creation of MBTs due to increased tortuosity (DCI >0.45). MBTs were established in 25 patients (8 of them underwent DS). The primary outcome was achieved in 16 patients receiving MBTs compared to 1 in the DS group. Implementation of an ECMO was necessary for four patients in the MBTs group (due to hemodynamic instability in two and shunt thrombosis in two) and none in the DS group.

**Table 2:** DS vs MBTs in patients with ductal curvature index >0.45.

**Description**	MBTs^**^	**MBTs**	**DS**	**OR**	**95%*CI***	***P*** **-Value**
Number	25^*^	17	15			
**Primary outcome**						
Hospital deaths	7 (28%)	4 (23.5%)	1 (6.6%)	3.5	0.4–28	0.2
Surgery/PVP/RVOT-stent	1	0	10	3	1.5–6	***0.000***
Permanent complication	1	1	1	0.8	0.05–15	0.7
**Secondary outcome**						
Need of ECMO	4 (16%)	3 (17.6%)	0	0.8	0.66–1	0.13
Primary outcome	16 (64%)	12 (64.7%)	3 (20%)	3.5	1.2–10	***0.005***
Impaired outcome	9^*^ (36%)	5^*^ (35.3%)	12^*^(80%)			

*OR, odds ratio; CI, confidence interval; DS, ductal stenting; MBTs, modified Blalock-Taussig shunt;*

** *includes patients receiving DS pre-operative, while these patients were excluded from the MBTs group. Statistical analysis was done between MBTs and DS. PVP, pulmonary valve perforation; RVOT, right ventricular outflow tract*.

* *in each of the two groups is one patient, who died due to non-DS/-MBTs related problems*.

### PA-IVS With RVDCC and Outcome

A total of five patients undergoing DS have completed the primary outcome while all (*n* = 6) patients in MBTs had impaired outcomes (Table 3). Of these two patients with DS needed MBTs to improve the pulmonary perfusion and one needed an unplanned Glenn anastomosis. The hospital deaths tallied three with MBTs and one in the DS group. The ECMO support was necessary for three patients with MBTs, while no ECMO was reported in the patients with DS. The major complications were related to DS in two and MBTs (low cardiac output with a need for ECMO in three and thrombosis in the right pulmonary artery with pericardial tamponade in 1) in four patients.

**Table 3:** DS vs. MBTs in patients with PA-IVS with coronary sinusoids.

**Description**	**DS**	**MBTs**	**OR**	**95%*CI***	***P*** **-Value**
PA IVS coronary fistel	7	6			
**Primary outcome**					
Need for unplanned shunt	2 (28.5%)				
Permanent complication	0	1^**^			
Hospital death	0	3 (50%)	0.5	0.2–1.1	***0.046***
Late death (before Glenn)	0	2^*^			
**Secondary outcome**					
Major complications	2 (28.6%)	4 (57.1%)	5	0.5–53	0.2
Need of ECMO	0	3 (50%)	0.5	0.2–1.1	***0.046***
Primary outcome	5 (71.4%)	0			
Impaired outcome	2 (28.6%)	6 (100%)	3.5	1–11.2	***0.004***

*OR, odds ratio; CI, confidence interval; DS, duct stenting; MBTs, modified Blalock-Taussig shunt; PA-IVS, pulmonary atresia with intact ventricular septum*.

* *one patient died due to cardiogenic shock 3 months after the operation, and the second died due to tumor-related problems*.

** *this patient could not be operated on due to shunt-related severe neurologic complications*.

## Discussion

All published comparisons between MBTs and DS were performed regardless of the complexity of ductal morphology (10–13). In a previous study published in 2021 (9), the results of our center showed that the patients with an increased ductal tortuosity and ductal curvature index (equal to or more than 0.45) have to be classified as the high-risk patients for duct stenting. The primary outcome in these patients was achieved in only 20% of them. However, the outcome in the patients from the same group who underwent MBTs is still unknown and the preferable palliative approach for this group is still controversial. In the current era, we have chosen first to establish a general comparison between the two procedures (DS and MBTs) regardless of the ductal morphology in the DS cohort and, secondly, to compare the outcome, more specifically in the two high-risk groups with PA and DDPC. The first group includes the patients with a high DCI >0.45, and the second includes patients with PA-IVC and coronary sinusoids in which the best palliative strategy seems to be controversial. For reasons of consistency, we have only included patients with a single lung supply (shunt or stent) in the analysis without concomitant additional repairs.

### General Comparison Between MBTs and DS

**Figure 1:**
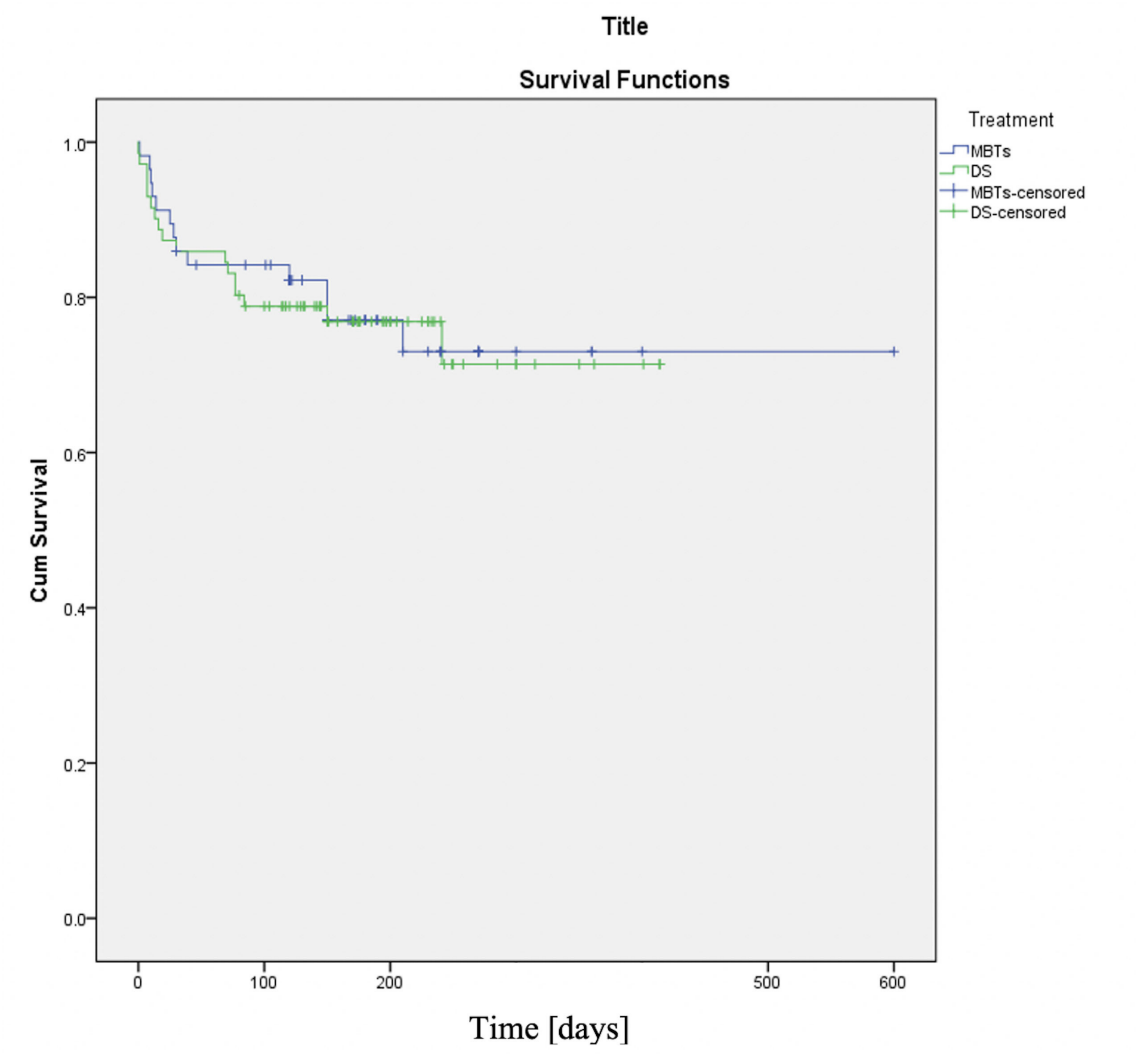
Kaplan-Meier curve (DS vs. MBTs). The two procedures showed the same decay over the whole population while reaching the primary endpoint. X-axis time since procedures is indicated in days.

The primary outcome of this study was achieved equally in the two procedure groups (77% in DS and 75% in MBTs), and no significant difference was observed. The survival analysis demonstrated no dissociation between the two procedures (Figure 1). The hospital deaths were dominated by patients who underwent MBTs (14.2%) compared to only 2.8% in the DS group (*P*-value 0.017). The same observations were documented in previous studies, which compared the two palliative strategies (11, 14). The high mortality rate in the MBTs group largely results from hemodynamic instability associated with diastolic run-off through the shunt and coronary artery steal (11), this phenomenon was observed in 75% of hospital deaths, while 25% were related to shunt occlusion. It is also worth noting that all patients who died following operations were on ECMO. The need for unplanned reoperation showed no significant differences between the two groups. The unplanned surgery in the DS group was required to treat desaturation related to stent failure or secondary to failure of ductal recanalization after acute ductal occlusion during the intervention. The unplanned surgeries in the MBTs group had to be performed either to treat a hemodynamic significantly pulmonary overload using shunt clips or to improve the saturation and the development of the pulmonary arteries by the creation of a large shunt (5 mm).

The major complications and need for ECMO were observed pre-dominantly in MBTs due to post-operative hemodynamic instability, shunt thrombosis, ventricular tachycardia, pericardial effusion, multiorgan failure related to a low cardiac output, and neurologic complications (intracranial bleeding and seizures). In contrast, the major complications observed in the DS groups were acute in-stent-thrombosis, stent dislocation, and vessel damage with the secondary severe neurologic complication. Alsagheir et al. (14) showed that DS demonstrates lower mortality and a lower risk of procedural complications. This observation is in agreement with the results of the present study.

The higher number of hospital deaths, complications, and need for ECMO in MBTs have made DS in many centers around the world the preferable palliative strategy in patients with DDPC. Contrary to the results of the previous studies, the current results showed that the need for PA-plasty was higher in the DS group. This can be explained by the highly tortuous ducts associated with a resulting functional pulmonary stenosis in our DS cohort (9).

### MBTs vs. DS in High-Risk Patients for Stenting (DCI >0.45)

**Figure 2:**
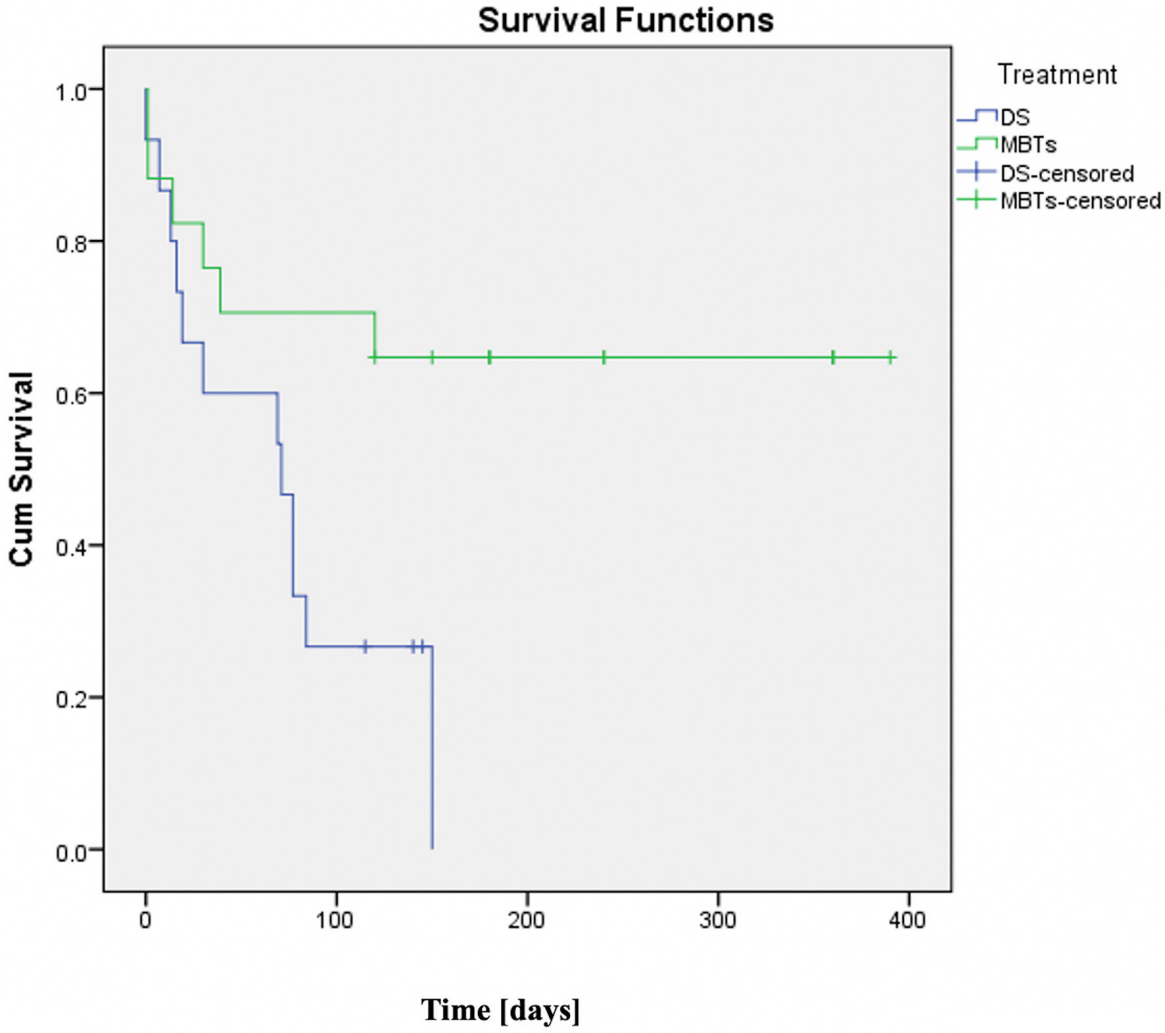
Kaplan–Meier curve (DS vs. MBTs in ducts with ductal curvature index (DCI) >0.45). Compared with DS-group with DCI >0.45 which showed very low survival, patients with a DCI >0.45 underwent MBTs and had much better survival rates with the majority reaching their next planned surgery. Time since procedures is indicated in days.

Although 64% of the patients in the MBTs group have achieved the primary outcome compared to only 20% in the DS group; the mortality and the need for ECMO in MBTs remained dominant compared to DS in this high-risk group. The survival analysis in Figure 2 demonstrates a significant difference between the two groups. DS seems inappropriate to bridge these patients to the next planned surgery without the need for additional operation.

It is important to note that a significant portion of hospital deaths in the MBTs group (57%) happened in patients who underwent DS and MBTs performed as an emergency either due to desaturation or after reanimation related to an acute ductal occlusion happened during the intervention. The last observation suggested that ductal stent failure in tortuous ducts might have a negative impact on the outcome of unplanned MBTs and make the surgery more challenging in these patients due to pre-operative desaturation and its related negative changes in pulmonary arteries and the lungs.

### MBTs vs. DS in Patients With PA-IVS and RVDCC

**Figure 3:**
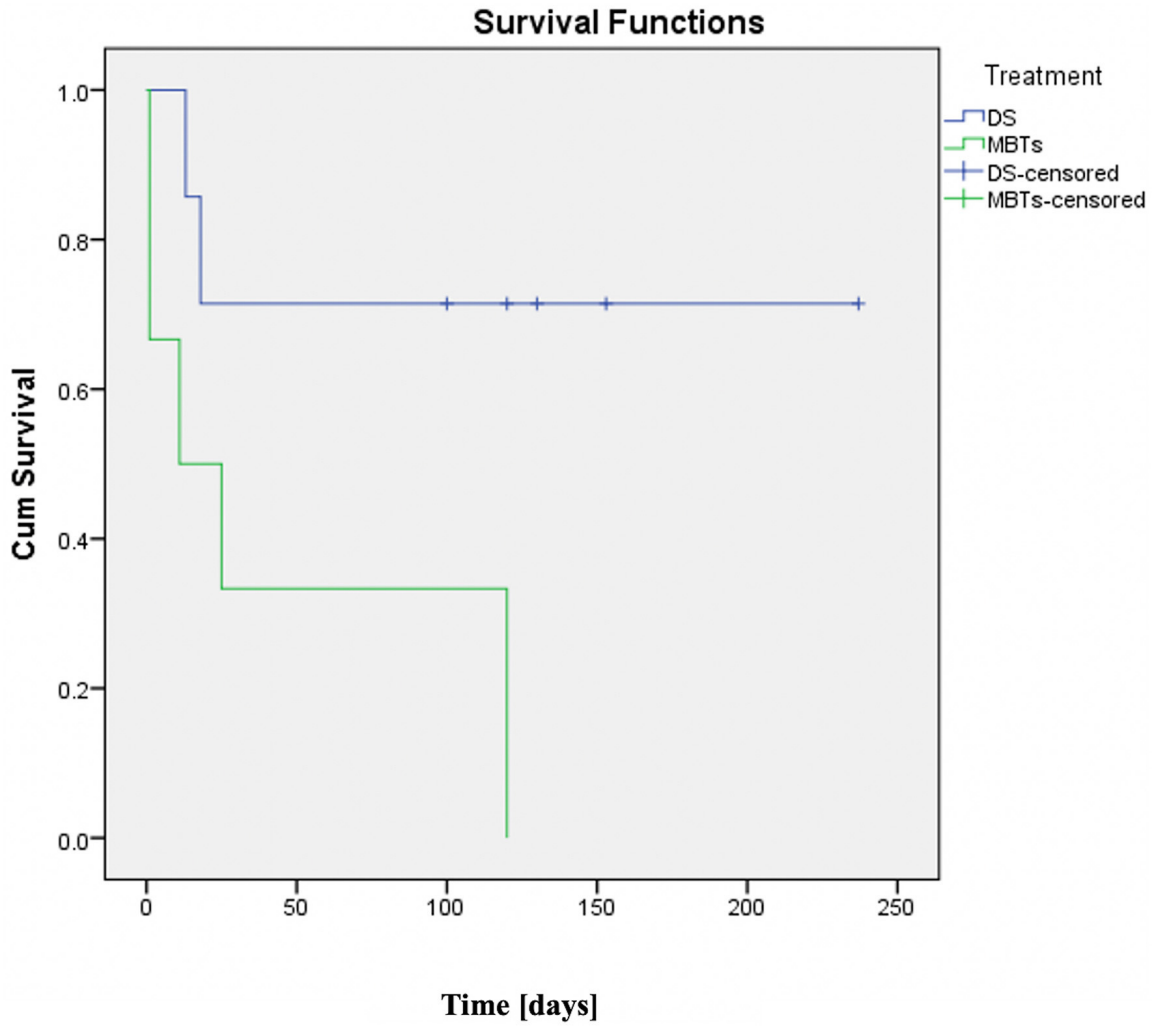
Kaplan-Meier curve (DS vs. MBTs in patients with PA-IVS and right ventricle-dependent coronary perfusion). DS group showed better survival rates compared with the MBTs group which had very low survival. No patient in this group has reached the next surgery. Time since procedures is indicated in days.

Although there was repeated interest to find out the solutions for patients with PA-IVS with RVDCC over the last decade (15, 16), there was no comparison published evaluating the two palliative procedures. We found that 57.2% of the patients in the DS group achieved the primary outcome compared to only 16.7% in the MBTs group. The use of cardiopulmonary bypass during cardiac operation seems not to have an impact on the outcome (four patients were operated on with bypass initiated *via* aortic and right atrial cannulation and two without it). The survival analysis (Figure 3) demonstrates a clear dissociation between the two palliative strategies. Hospital deaths and the need for ECMO were reported in half of the patients who underwent MBTs. The post-operative documented significant ST changes and ventricular arrythmia, decreased ejection fraction of the single ventricle due to ischemia and diastolic run-off through the shunt and coronary steal can explain the higher mortality and morbidity in these patients compared to those undergoing DS. There were no significant differences in complications between the two groups (Table 3).

## Conclusion

With low mortality and morbidity, the DS is an alternative palliative strategy to be considered in relation to MBTs to secure the pulmonary blood flow in patients with PA and DDPC. It can improve the symmetrical growth of the pulmonary arteries. This advantage seems less dominant in stenting the tortuous ducts with a DCI of more than 0.45 (9).

Contrary to the previous result, this study suggests that the MBTs could be preferred to DS in patients with DCI higher than 0.45. However, the hospital deaths remain dominant in the MBTs while the DS seems to be suitable for the palliative strategy in the patients with PA-IVS and RVDCC by decreasing the risk of intra- and post-operative ischemia and ventricular arrhythmia observed.

## Data Availability Statement

The raw data supporting the conclusions of this article will be made available by the authors, without undue reservation.

## Ethics Statement

Ethical review and approval was not required for the study on human participants in accordance with the local legislation and institutional requirements. Written informed consent from the participants' legal guardian/next of kin was not required to participate in this study in accordance with the national legislation and the institutional requirements. Written informed consent was not obtained from the minor(s)' legal guardian/next of kin for the publication of any potentially identifiable images or data included in this article.

## Author Contributions

NM: conception and design of the work and data collection. NM and PZ: data analysis and interpretation and drafting the article. MS, BA, and PZ: critical revision of the article. NM, MS, BA, MM, and PZ: final approval of the version to be published all authors. All authors contributed to the article and approved the submitted version.

## Conflict of Interest

The authors declare that the research was conducted in the absence of any commercial or financial relationships that could be construed as a potential conflict of interest.

## Publisher's Note

All claims expressed in this article are solely those of the authors and do not necessarily represent those of their affiliated organizations, or those of the publisher, the editors and the reviewers. Any product that may be evaluated in this article, or claim that may be made by its manufacturer, is not guaranteed or endorsed by the publisher.

## Abbreviations

PDA: patent ductus arteriosus
DCI: ductal curvature index
PA: pulmonary atresia
CI: confidence interval
mBT shunt: modified blalock-taussig Shunt
RVDCC: right ventricle-dependent coronary circulation.
